# The Epithelial Immune Response to Human Papillomavirus Infection

**DOI:** 10.3390/pathogens14050464

**Published:** 2025-05-09

**Authors:** Shyantani Roy-Biswas, Merilyn Hibma

**Affiliations:** Department of Pathology, Dunedin School of Medicine, University of Otago, P.O. Box 56, Dunedin 9054, New Zealand; roysh670@student.otago.ac.nz

**Keywords:** human papillomavirus, innate immunity, adaptive immunity, disease regression, disease progression, HPV immune evasion, cervical intraepithelial neoplasia

## Abstract

The skin is a complex organ, containing an intricate network of immune cells that are crucial for host barrier function and defence against pathogens. Human papillomavirus (HPV) exclusively infects the skin, and its lifecycle is intimately associated with epithelial cell division and differentiation. There are over 450 HPV types, 12 of which are classified as carcinogenic. The primary focus of this review is the epithelial immune response to HPV infection of the cervix during the initial stages of infection, productive infection, and disease progression. During the early stages of infection, cells are HPV-positive; however, there are no attributable histological changes to the epithelium. The HPV-infected cells have the capacity for innate sensing and signalling through toll-like receptors in response to viral nucleic acids. However, HPV has evolved multiple mechanisms to evade the innate response. During productive infection, all viral antigens are expressed and there are visible histological changes to the epithelium, including koilocytosis. Disease regression is associated with Tbet positive cells in the infected epithelium and the presence of CD4 and CD8 T cells in the lamina propria. Disease progression is associated with the overexpression of the E6 and E7 oncoproteins after integration of viral genomes into the host chromosomal DNA. Histologically, the epithelium is less differentiated, and changes to cells include a higher nuclear-to-cytoplasmic ratio and an increased mitotic index. Immune changes associated with disease progression include increased numbers of cells expressing suppressor molecules, such as FoxP3, Blimp-1, and HMGB1, and myeloid cell infiltrates with an M2-like phenotype. This review highlights the gaps in the understanding of the immune response in HPV-positive cervical neoplasia, and in regression and progression of disease. This knowledge is critical for the development of effective immunotherapies that reliably cause HPV-positive cervical neoplasia to regress.

## 1. Human Papillomavirus Infection

This article is a narrative review of the literature that focuses on the epithelial response to human papillomavirus (HPV) infection. While HPV causes infections and is oncogenic at various sites in humans, the primary focus of this review is the epithelial immune response to cervical infection and HPV-positive cervical neoplasia. Subject-specific searches were carried out for the topics covered in this review using PubMed, Scopus, Web of Science, and Google Scholar or Google. While the review focuses on disease of the cervix, many of the concepts are generalisable to other HPV-associated diseases, and in some cases, data from HPV infections at other sites or animal models are included.

HPVs are small, non-enveloped, double-stranded DNA viruses that infect human cutaneous and mucosal epithelium. There are about 450 different HPV types, divided into five genera: alpha-(α), beta-(β), gamma-(γ), mu-(μ), and nu-(ν) papillomaviruses. The α-papillomavirus genus contains HPV types tropic for mucosal epithelium, including oral, rectal, vaginal, and cervical mucosa [1].

HPV has a long infectious cycle of at least three weeks. The entire lifecycle occurs within stratified, differentiating epithelium, where viral genes are exclusively expressed (reviewed in [1]). HPV binds to the basement membrane and infects the epithelial basal cells, exposed following abrasion or trauma to the epithelium. In mitotically active cells, the expression of the viral proteins is controlled by the p97 promoter. Early genes such as E6 and E7 are expressed only at low levels [2,3]. The viral differentiation-dependent promoter is activated when epithelial cells differentiate into mature keratinocytes, increasing viral gene expression [4].

HPVs do not encode their own replication factors, instead they utilise the host cell’s replication machinery [1]. Viral replication and assembly occur at subnuclear structures known as promyelocytic leukaemia (PML) nuclear bodies [5]. The viral differentiation-dependent promoter is turned on when epithelial cells differentiate into mature keratinocytes, increasing viral gene expression [4]. All viral genes, including those encoding for the late L1 and L2 proteins, are expressed in the upper differentiated layers of the stratum spinosum and granulosum of squamous epithelia. Virus assembly occurs in these upper layers, and the assembled infectious virions are shed in intact squames [6].

## 2. Human Papillomavirus and Disease

Most papillomaviruses cause asymptomatic, self-limiting, or latent infections; however, some HPV types are associated with an increased risk of cancer at specific sites in the body [7]. HPV types are characterised as low- or high-risk, based on their oncogenic potential. Low-risk mucosal types, such as HPV6 and 11, cause benign genital warts whereas high-risk types, such as HPV16 and 18, may cause high-grade neoplasia and cancer at sites including the cervix, anus, vagina, oropharynx, vulva, and penis [8].

Viral DNA is maintained as episomes in cells; however, integration into the host chromosomal DNA occurs in HPV-neoplasia and cancer and this can occur with the continued maintenance of episomes in those cells. Following integration of the viral genome, E2 repression of the p97 promoter is lost and E6 and E7 expression is increased. E6 binds and degrades p53, whereas E7 binds to pRB, contributing to cell cycle dysregulation. Doorbar and Griffin (2019) have proposed that non-productive HPV infection in the columnar epithelium close to the squamocolumnar junction of the cervix is associated with dysregulated gene expression that directly results in high-grade cervical neoplasia (CIN), providing an explanation for the increased cancer susceptibility at the transformation zone of the cervix [9].

Based on cervical cancers diagnosed between 2005 and 2015 in Australia, around 93% of cervical cancers are HPV-associated [10]. A systematic review of 1174 studies up to March 2024, including data from 121 countries, reported that HPV16 is the most prevalent type detected in HPV-positive invasive cervical cancer (61.7% of cases), followed by HPV18 (15.3% of cases), while HPV45, 33, 58, 31, and 52 are the other major contributing types, with a further 10 HPV types identified that are less frequently represented [11]. Other HPV-associated cancers include vulval, vaginal, anal and penile cancers [12]. High-risk HPV types also contribute to HPV-positive head and neck squamous cell carcinoma (HNSCC), which is increasing in incidence in the USA (2.7% increase in men and 0.8% in women during 1999–2015) [12]. Around 70% of cases of HNSCC diagnosed between 1993 and 2005 are HPV-positive in the USA [13]. HPV16 (84%) was the dominant type found in 73 HPV-positive HNSCC identified on The Cancer Genome Atlas, whereas HPV18 was not detected [14].

## 3. The Immune Landscape in the Normal Cervix

The mucosa of the genital tract consists of the squamous epithelium (type II mucosa) in the lower genital tract and the type I columnar mucosal epithelium in the upper genital tract. The transformation zone at the cervix is the site of the transition between these two mucosal types and is where cervical cancer most frequently occurs.

The cells of the cervix have been defined by single cell sequencing using biopsy samples and resection specimens of HPV-positive, cytology normal, low (LSIL) and high (HSIL) grade squamous intraepithelial lesions, and cervical cancer tissues [15,16,17]. In a combined analysis of these samples, 35% of the cells were structural and included epithelial cells, fibroblasts, endothelial, and smooth muscle cells [15]. The remainder are immune cells and include predominantly natural killer (NK) and T cells, followed by neutrophils, myeloid cells, mast cells, B cells, and plasma cells [15].

As might be expected, exfoliated samples have a higher representation of immune cells compared with biopsy specimens [18]. In exfoliated samples, neutrophils, and also T cells, myeloid cells, mast cells, NK cells, B cells, and plasmacytoid dendritic cells (pDC) predominate [18]. However, it is important to note that the proportion of HSIL and cervical cancer cells was greater relative to LSIL and normal in the sequenced samples compared to the study by Guo et al. (2023) [15], and that there was variability between the samples that were tested.

## 4. Cells in the Tissue Microenvironment in Early-Stage Papillomavirus Infection

**Figure 1:**
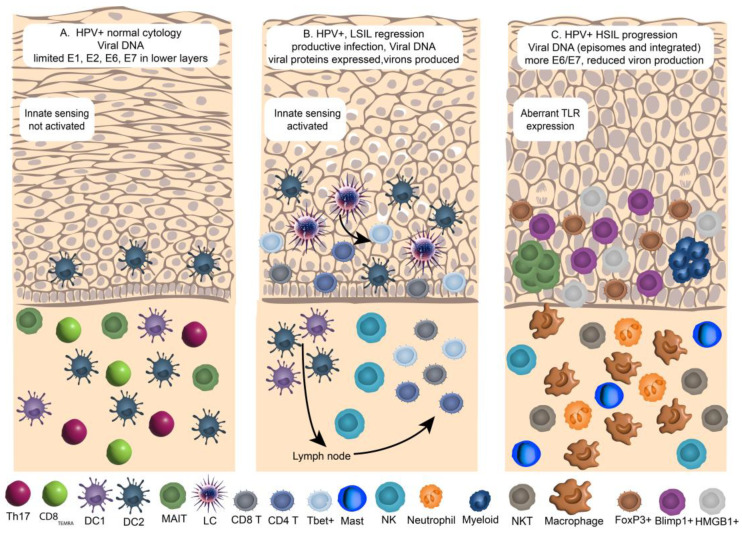
A schematic representation of the differences in the immune microenvironment in the following: (**A**) HPV-positive normal cytology. Based on single cell sequencing data, cDC2 dominate over cDC1 cells and there are increased numbers of MAIT cells, Th17 cells, and CD8+ T_EMRA_ cells in HPV-positive tissues with normal cytology when compared with normal cervical epithelium. (**B**) Productive infection and LSIL. Innate sensing contributes to the triggering of a viral antigen-specific T cell response in regressing lesions. The presentation of viral antigens to local T cells by LCs and DC2s and to lymph node T cells by DC1 and DC2 occurs. Infiltration of CD4 and CD8 T cells into the lamina propria and Tbet+ cells in the epithelium is associated with regression. NK cells are increased in LSIL. (**C**) Progressed disease and HSIL. Increased numbers of cells expressing suppressor molecules including Blimp-1, FoxP3, and HMGB1, immune inhibitory clusters of MAIT cells, and immune surveillence clusters of myeloid cells and also M2-like macrophages are associated with progression. Neutrophils are inversely correlated with CD8 T cells and may be inhibiting their function. The figure was drawn in Adobe Illustrator v. 29.5. The LC is AI-generated using Image Playground (MacOS 15.4) and some cells were provided by Dr Mayumi Saito.

The changes to the tissue microenvironment in early-stage infection are summarised in Figure 1A. During the early stages of infection, tissues are HPV-positive and histologically normal. Viral DNA is present, and protein expression is limited to low levels of proteins that support episomal replication in the infected basal keratinocytes. The changes to cells in the tissue microenvironment in this initial phase of infection identified by single cell sequencing include an increase in conventional cDC2 cells and a relative drop in cDC1 cells in HPV-positive lesions compared with HPV negative tissues [15]. Of the innate cell subsets, Mucosal-associated invariant T (MAIT) cells were proportionally increased in the HPV-positive tissues, relative to other CD8+ T cell subsets. The notable differences in T cell populations that were identified between HPV-positive and negative cytology samples included increased representation of Th17 cells and CD8+ T_EMRA_ cells [18]. The CD8+ T_EMRA_ subset had high *CX3CR1* expression and a *PRF1* and *KLRD1* positive cytotoxicity signature.

## 5. The Innate Response to HPV Infection

There is a dependence on innate sensing during the initial phases of infection due to the limited viral protein expression. Keratinocytes have the capacity to respond to infection using a range of innate sensing mechanisms including Toll-like receptors (TLRs), sensing through cGAS-STING and AIM2 and the interferon (IFN) response through the activation of IFN-stimulated response elements (ISREs), and the expression of IFN-stimulated genes (ISGs) following binding of IFN to its receptor (reviewed in [19]). Additionally, free virions and intercellular signalling may activate other uninfected cells, including immune cells at the infection site. Innate recognition of HPV pathogen-associated molecular patterns (PAMPs) is critical for the initiation of the adaptive response. The initial low levels of HPV PAMPs and innate recognition may contribute to a delay or absence of immune-mediated regression of HPV infection.

### 5.1. Innate Sensing in Papillomavirus Infection

#### 5.1.1. Toll-like Receptors

Innate sensing by TLRs is crucial in shaping the host response to infection. TLRs are membrane proteins that bind to molecular patterns, including lipopolysaccharides, double-stranded RNA, and flagellin from bacteria, fungi, and viruses. Following binding of PAMPs to TLRs, a signalling cascade is triggered with the recruitment of adaptor molecules, followed by the activation of transcription factors and the production of proinflammatory and immune-mediated cytokines that contribute to an antiviral response [20]. TLR activation can also induce molecules, such as COX-2, that can inhibit the antiviral response [21].

Of the 13 TLRs identified in mammals, TLRs 1-10 are expressed in humans. TLRs 1, 2, 4, 5, and 6 are located on the cell surface, whereas TLR3, TLR7, TLR8, and TLR9 are localised intracellularly in endosomes or in the endoplasmic reticulum. Among the TLRs that are primarily involved in sensing, viral nucleic acids, TLR3 is activated by double-stranded RNA [22], TLRs 7 and 8 are activated by single-stranded RNA [23], and TLR9 is activated by double-stranded CpG-rich DNA [24]. While the remaining TLRs are primarily involved in bacterial sensing, some recognition of viral components has also been reported. For example, TLR4 is activated by lipopolysaccharides but can also be activated by some glycosylated viral structural and non-structural proteins [25,26]. TLR1 and TLR6 form heterodimers with TLR2 and primarily recognise bacterial and mycoplasma lipoproteins [27,28,29]; however, TLR2 also activates innate immunity to viruses, including respiratory syncytial virus and vaccinia virus [30,31]. TLR5 is stimulated by bacterial flagellin [32] but can be upregulated following viral infection [33].

Several studies have assessed typical TLR expression in cervical cells. In one study, the expression of TLRs was measured in short term cultures of normal endo- and ectocervical cells [34]. TLRs 2, 3, 5, and 6 were most abundantly expressed, while TLR1 and 2 were expressed at lower levels in cultured endocervical cells. The expression pattern in ectocervical cells was similar, with reduced levels of TLR2 and 5. TLR7, 8, and 9 were expressed only at low levels in endo- and ectocervical cells. The relative expression of TLRs in the epithelium and stroma of normal cervical samples has also been assessed [35]. TLRs 1–5 were more highly expressed in the epithelium compared with TLRs 6–9. TLRs 1–5 were very highly expressed in the stroma and the expression of TLRs 6, 7, and 9, but not TLR8, were also increased in the stroma relative to the epidermis.

In a study of HPV-positive disease, mRNA levels of *TLR2*, *3*, *4*, and *8* were assessed in cytology samples. *TLR3* expression was suppressed in HPV-positive LSIL and HSIL, whereas *TLR2*, *4*, and *8* expressions were increased in HPV-positive LSIL relative to the controls [36]. Rice et al. (2023) performed immunohistochemical analysis of TLRs 1, 4, 7, and 8 in five LSIL and five HSIL samples, comparing staining in lesional tissue to the surrounding epithelium and stroma [37]. TLR4 and 8 were not expressed in the lesional or adjacent epithelium. TLR1 was absent in adjacent epithelium but was upregulated in LSIL and HSIL. TLR2 was expressed in adjacent epithelium and further increased in LSIL and HSIL. TLRs 3 and 7 were weakly to moderately expressed in adjacent epithelium and notably increased in LSIL and HSIL, as was retinoic acid inducible gene-I (RIG-I) [37]. The lack of expression of TLR4 in HSIL contrasts with Santoro et al. (2024), who reported positive, and in some cases strong, TLR4 staining in HSIL using a different antibody [38]. Overall, reports on TLR expression in LSIL are inconsistent, and further rigorous studies with validated antibodies and larger sample sizes are needed to clarify TLR expression in these tissues. This knowledge is critical to inform the use of TLR agonists in HPV-associated disease.

Daud et al. (2011) investigated the association between TLR expression in tissues and viral clearance or persistence in women with incident infections with HPV16 or HPV51 [39]. The increased expression of TLRs 2, 3, 7, 8, and 9 was associated with clearance of HPV16 but not HPV51. In contrast, persistence was associated with a lack of increased TLR expression [39]. In another study, regression of CIN 2 was associated with high levels of TLR2 and TLR7 [40]. Scott et al. (2015) examined the expression of TLR2, 3, 7, 8, and 9 in Cytobrush samples from women with high-risk HPV-positive incident infections in relation to IFN-γ ELISpot responses to E6 and E7. Higher levels of the expression of TLR3, 7, 8, and 9 were correlated with a peripheral E6-specific ELISpot response but not with an E7-specific response [41].

Functionally, the TLR-receptor adaptor molecule 1 (TICAM1), a key molecule in the TLR3 pathway mediating NF-κB and IFN regulatory factor (IRF) activation, is not upregulated following poly(I:C) stimulation of primary foreskin keratinocytes containing HPV16 or HPV18 episomes [42]. The authors suggest that the dampening, but not complete blocking, of pattern recognition receptor signalling may delay recruitment of innate immune cells to the site of HPV infection.

HPV PAMPs include double-stranded viral DNA, single-stranded RNA, and viral proteins. The association between increased TLR expression and disease regression suggests that the TLRs are being bound by papillomavirus PAMPs, activating an immune response. Co-culture of murine bone marrow-derived dendritic cells with HPV16 L1-containing virus-like particles stimulates MyD88, indicating that TLR recognition of HPV particles at least occurs in vitro [43]. Infection with other viruses, such as human cytomegalovirus (CMV), also increases the expression of TLRs such as TLR4 and 5, possibly through indirect immunomodulatory or immunostimulatory effects [33].

Interestingly, the TLRs upregulated in LSIL and HSIL extend beyond those known to be activated by HPV PAMPs. Given the complexity of the cervix as a mucosal site and its intimate association with the microbiome, it is plausible that the increased TLR expression results from microbiome-driven immune activation rather than a direct response to HPV. In this context, a recent clinical trial of an E6 peptide immunotherapy (PepCan) showed that the *C. albicans* antigen control arm performed better than the HPV16 E6 peptide combined with the *C. albicans* antigen in regressing CIN 2 and 3 [44]. *C. albicans* activates TLRs primarily through TLR2 and 4. Indeed, several TLR agonists, including *C. albicans* and Imiquimod (a TLR7 agonist), have been used to trigger immune-mediated regression for the treatment of genital and common warts [45,46,47].

#### 5.1.2. Other Innate Sensors

Other DNA sensors include AIM2, which is activated by cytosolic DNA and triggers inflammasome formation and IL-1β and IL-18 release [48,49]. AIM2 is not normally expressed in keratinocytes but is highly expressed in Langerhans cells (LCs) in normal skin, and its expression is markedly increased in HPV16 positive anal intraepithelial lesions [50]. Activation of the AIM2 inflammasome upon detection of HPV16 DNA in keratinocytes leads to the release of IL-1β and IL-18. However, in the context of HPV infection, AIM2 is required for the suppression of IFN-β activation [51].

IFN-γ-inducible protein 16 (IFI16) is another cytosolic DNA sensor that detects double-stranded viral DNA and triggers IFN-β production, activating a potent antiviral response through the Janus Kinase/Signal Transducer and Activator of Transcription (JAK/STAT) pathway and the induction of ISGs [52,53]. STAT1 and STAT2 drive IFN-mediated immune signalling that inhibits cellular proliferation and viral replication. For example, the ISG IFN-induced protein with Tetratricopeptide Repeats 1 (IFIT1) inhibits the HPV E1 helicase, preventing viral DNA amplification [54]. HPV can also regulate STAT1, inhibiting the IFN response [55]. IFN receptor binding stimulates the JAK/STAT pathway, triggering ISG expression. High-risk HPV types inhibit IFN-κ expression via E6 and E7, altering the expression of pathogen receptors, ISGs and their downstream cytokines and chemokines [56,57]. Furthermore, HPV can downregulate specific ISGs. For example, HPV16 E6 expression in human keratinocytes inhibits IRF6 transcription, reducing the IL-1β promoter activity and blocking IL-1β production [51,58,59].

The cyclic GMP-AMP synthase (cGAS)-stimulator of IFN genes’ (STING) signalling pathway is also important for double-stranded DNA sensing. cGAS is located in the cytoplasm and in the nucleus, where it is bound to chromatin [60]. Upon binding dsDNA, cGAS catalyses the synthesis of cyclic GMP-AMP (cGAMP), which is then sensed by the ER membrane protein STING. STING oligomerises and relocates to the Golgi, where it is phosphorylated by TBK1. This leads to IRF3 phosphorylation, translocation to the nucleus, and induction of type 1 IFNs, ISGs, and NF-κB.

The HPV18 oncoprotein E7 inhibits STING to suppress the cGAS pathway [61]. However, in primary foreskin keratinocytes containing HPV16 or HPV31 episomes, cGAS is upregulated while STING levels remain unchanged. E6 is required for cGAS upregulation, and this is p53-dependent. The cGAS-STING pathway remains functional in HPV-positive cells, as evidenced by type I IFN expression following cGAMP treatment. The knockdown of cGAS does not affect episome maintenance or viral transcription [62]. HPV-positive cells are sensitised to DNA damage-induced apoptosis via cGAS, increasing their sensitivity to DNA-damaging drugs [62].

There is ongoing debate over whether cytoplasmic DNA sensors are exposed to HPV DNA during infection. Uhlorn et al. (2020) showed that the transfection of HPV DNA stimulates strong cGAS-STING responses. However, during natural infection, HPV is contained in a vesicular trafficking pathway that shields it from cGAS-STING detection [63]. It remains possible that HPV DNA becomes exposed to the cytosol during cell division.

DNA sensors trigger either type I (α or β) or type III (γ) IFN responses, or activate inflammasome-related cytokines, including IL-1β and IL-18. Secretion of these cytokines by infected keratinocytes in response to innate sensing attracts immune cells to the infection site and contributes to the initiation of the adaptive immune response [64,65,66]. However, following the integration of the HPV genome into the chromosomal DNA of the infected keratinocytes, IL-1β secretion is blocked [42].

### 5.2. Innate Cells in Papillomavirus Infection

Innate cells bridge innate and adaptive immunity, enabling the activation of the adaptive immune response, enhancing protection and contributing to the establishment of long-term memory. Evidence from single cell sequencing of HPV-positive, cytology-negative tissue has identified innate cell subsets that have not been extensively studied in the context of HPV infection. While some of these innate cells, such as Natural Killer T (NKT) cells and NK cells, are known for their antiviral effects, there is emerging evidence supporting antiviral functions for other cell types, including mast cells, neutrophils, and MAIT cells. This highlights the need for a better understanding of the functions of these cell subsets at the site of HPV infection and in HPV-associated disease progression.

#### 5.2.1. NKT Cells

NKT cells are characterised by the expression of the invariant T cell receptor (TCR), also known as the invariant antigen receptor. The type I NKT cells, or invariant NKT (iNKT) cells, recognise lipid antigens including α-GalCer, presented by CD1d. Type II NKT cells, also known as non-invariant NKT cells, have a more diverse invariant TCR, are also CD1d-dependent, but are not reactive against α-GalCer. In response to TCR ligation, NKT cells produce proinflammatory T helper 1 (Th1) cytokines, including IFN-γ and tumour necrosis factor (TNF), as well as anti-inflammatory Th2 cytokines such as IL-4 and IL-10 [67].

An important function of NKT cells is engagement with CD40 on DCs via CD40L to induce full DC maturation [68]. iNKT cells can also produce IFNγ in response to α-GalCer and can trigger IFNγ production by DCs [68,69]. In the K14 E7 transgenic mouse, rejection of HPV16 E7-expressing skin is inhibited by NKT cells that are attracted to the site, through an IFNγ-dependent mechanism [70]. Additionally, and independent of IFNγ, NKT cells reduce the capacity of DCs to present antigen to T cells in the draining lymph nodes, suppressing the generation of an antigen-specific T cell response to skin-expressed HPV16 E7 [71]. iNKT cells are increased in high-grade CIN and IFNγ expression is higher in HPV-positive lesions, suggesting that the suppression of T cells by local iNKT cells via IFNγ may also occur in HPV disease in the cervix [72].

#### 5.2.2. NK Cells

NK cells are large granular CD56+ cells that recognise targets with downregulated MHC class I. Under normal physiological conditions, MHC class I molecules on neighbouring cells engage NK cell inhibitory receptors, including NKG2A and KIR, maintaining NK cells in an inactive state [73]. NK cells are activated in the absence of inhibitory receptor engagement, following the loss of MHC class I on target cells [74]. NK cell activation can also occur through ligand binding to NK cell activating receptors, such as NKG2D. However, MICA and ULBP2, the ligands for the activation receptor NKG2D, are not expressed in CIN2/3 lesions. Similarly, CD155, the ligand for the activation receptor DNAM-1, is absent in CIN2/3 tissues [75].

Papillomavirus infection is a major indication of an underlying NK deficiency, suggesting that NK cells play a role in viral control [76]. Allogenic hematopoietic cell transplantation restored NK cell cytotoxicity and resulted in persistent remission of all HPV-related disease in an individual with *IL2RG*-mutant NK cells [77], further supporting a role for NK cells in the control of HPV. HPV-infected cells may be susceptible to NK cell attack because the early viral proteins, E5 and E6, downregulate surface MHC class I [78,79], although MHC class I expression on HPV lesions can be variable.

NK cells are broadly divided into two subsets: the CD56^bright^NK cells, which account for around 10% of the circulating and the majority of tissue-associated NK cells, and the CD56^dim^NK cells, which comprise the remaining circulating NK population [80]. CD56^bright^NK cells secrete cytokines, including IFNγ, TNF, GM-CSF, IL-10, and IL-13 [81]. CD56^bright^NK cells can develop into CD56^dim^NK cells, which are terminally differentiated, express high levels of perforin and granzyme, and exert cytotoxic effects [80,82].

CD56+ cells are found in CIN1–3 lesions and are significantly increased in the stroma of CIN 1 and 2 tissues compared to normal stroma [75]. NK cell numbers are also elevated in HPV16 but not HPV18 cytology samples [83]. Furthermore, individuals with HPV16 positive high-grade lesions have significantly increased numbers of CD56^bright^NK cells in the peripheral blood [84].

Although NK cell numbers are increased in CIN, their cytotoxic function and IFNγ transcript levels are reduced [83]. Zhang et al. (2019) found abundant CD16+ NK cells with increased IFN-γ secretion in HPV16 positive women [83]. However, these NK cells fail to eliminate mature HPV virus due to inadequate IL-2 production. IL-2 positively regulates NK cell cytotoxicity as well as virus-induced KLRG-1 expression [83]. In contrast, Renoux et al. (2011) also reported that NK (NKp46+) cells are increased in squamous intraepithelial lesions but showed that NK cells take up HPV VLPs in a CD16-dependent manner and that CD16 is required for cytotoxic activity and cytokine release in vitro. They suggested that NK cells could contribute to spontaneous regression of HPV-associated cervical neoplasia [85].

Mechanistically, IL-18-dependent IFNγ production by NK cells is inhibited by competitive binding of E6 and E7 to the IL-18 receptor. HPV-positive cell lines with high viral genome copy numbers integrated into the host DNA release E6 and E7 extracellularly, where they bind to IL-18 receptors on co-cultured NK cells, suppressing IL-18-dependent IFNγ production [86].

Several co-inhibitory receptors on NK cells, including TIGIT and KLRG1, further suppress NK cell function [87,88,89,90,91]. Nie et al. (2022) demonstrated significantly increased expression of poliovirus receptor (PVR), the ligand for TIGIT, as well as E-cadherin and N-cadherin, ligands for KLRG1, with increasing CIN grade in HPV16 positive disease. They also found that the CD56^bright^NK cells in the peripheral blood from these individuals had a significantly reduced ability to secrete IFN-γ. The upregulation of ligands for TIGIT and KLRG1 in the cervical tissues may therefore contribute to the functional impairment of CD56^bright^NK cells, promoting HPV16-associated CIN progression [84].

#### 5.2.3. Mast Cells

Mast cells are granulated cells that reside in nearly all tissues in the body. In the cervix, Guo et al. (2023) identified a population of mast cells that constituted 2.1% of the total cells single cell sequenced from normal HPV-positive cervix, high-grade disease, and cancers. They showed that the proportion of mast cells was highest in normal cervical tissue and decreased with increasing CIN grade, with the lowest proportion found in cervical cancer. Tissue staining revealed that mast cells were more frequently located in the stroma than in the epithelium and were present at twice the frequency in both compartments in CIN 2 and 3, compared with normal cervix [92].

Mast cells express a range of innate sensors, including TLRs, RIG-I, melanoma differentiation-associated protein-5 (MDA-5), and complement receptors. They also contain pre-stored mediators such as TNF and MMPs (reviewed in [93]). As sentinel cells, mast cells can secrete cytokines that recruit other immune cells to the site of infection. In the K14 E7 mouse model, mast cells are attracted to the skin by CCL2 and CCL5, which are secreted in response to HPV16 E7-induced epithelial hyperplasia [94].

Although mast cells are understudied in the context of HPV infection in humans, in herpes simplex virus (HSV) infection, keratinocytes produce the alarmin IL-33, which triggers mast cells to secrete TNF and IL-6, providing protection against cutaneous HSV [95,96]. It is therefore plausible that mast cells may contribute to disease regression, and that HPV may have evolved mechanisms to evade mast cell-mediated antiviral responses.

#### 5.2.4. Neutrophils

Neutrophils are short-lived granulocytes that are the predominant infiltrating cell type during acute inflammation. Upon stimulation with inflammatory signals, their programmed cell death is delayed [97].

Neutrophils mediate various antiviral affects. Together with macrophages, neutrophils can phagocytose virus-infected apoptotic cells [98]. They are activated through MDA5 and RIG-I to secrete IFN-β and TNF, contributing to viral clearance [99]. Neutrophils also produce antimicrobial agents such as myeloperoxidase (MPO) and α-defensins, which can inactivate viruses [100]. These molecules are incorporated into neutrophil extracellular traps (NETs) produced by neutrophils. NETs consist of net-like structures of genomic DNA containing the NET-bound antimicrobial proteins. NETs can trap and inactivate human immunodeficiency virus (HIV)-1 using NET-bound MPO and α-defensin [101].

Neutrophils can present antigen to CD8+ T cells, contributing to antiviral immunity [102]. In HPV infection and disease progression, an inverse correlation was observed between neutrophil and T cell numbers in lesions, suggesting that neutrophils may inhibit T cell activity. Supporting this, neutrophils in CIN lesions express low levels of CD62L, consistent with a suppressive phenotype. In vitro, neutrophils co-cultured with T cells inhibited T cell activation, proliferation and IFN-γ secretion [103].

#### 5.2.5. MAIT Cells

Mucosal-associated invariant T (MAIT) cells are a Vα7.2+ and CD161+ T cell subset that express the transcription factors RORγT and Tbet [104,105,106]. They are widely distributed throughout the genital tract. Endocervical MAIT cells are primarily located adjacent to the columnar epithelium. In the transformation zone, they are mainly in the lamina propria; in the ectocervix, they are found on both sides of the basement membrane [107].

MAIT cells recognise antigen presented by the invariant MHC-related molecule-1 (MR1) [108]. Although MR1 primarily binds Vit B Ag released by commensal microbes (e.g., bacteria) rather than viral antigens, its expression is downregulated by several viruses, including HSV via its US3 kinase [109]. This suggests that the loss of MR1 provides a biological advantage to viruses. MAIT cells are also activated in a TCR-independent manner by cytokines, including IL-12 and IFN-α (reviewed in [110]). Consequently, MR1-independent MAIT cells’ responses include proliferation, provision of B cell help, secretion of cytokines such as IFN-γ, TNF, and IL-2, and upregulation of perforin/granzyme and associated cytotoxicity [111,112].

MAIT cells also regulate other cells locally. For example, they negatively regulate NK cell maturation and NK cell-dependent anti-tumour immune responses under steady state conditions, while activated and expanded MAIT cells promote anti-tumour immunity via IFN-γ-dependent NK cell activation [113].

Despite their presence in the genital mucosa, the role of MAIT cells in HPV infection and disease progression is poorly understood. A significantly lower percentage of MAIT cells (within the CD3+ cell population) has been observed in the peripheral blood of patients with progressive cervical cancer compared to healthy individuals, with further reductions in advanced-stage disease [114]. However, a limitation of this study is the lack of tissue data and functional assessment. While some studies have associated increased MAIT cell infiltration in tumours with poor prognosis, others have reported improved survival outcomes (reviewed in [115]).

## 6. Productive Infection and the Adaptive Immune Response

The adaptive immune response plays an important role in the clearance of productive HPV infection. Most cases of low-grade squamous intraepithelial lesions or CIN 1 (the pathological manifestation of productive HPV infection) can be cleared and are associated with an adaptive immune response specific for viral antigens expressed in infected keratinocytes (see Figure 1B). In the absence of an effective immune response, for example, in immunocompromised patients, there is an increased incidence of low-grade cervical dysplasia in the anogenital tract [116].

### 6.1. Antigen Presentation and the Initiation of the Adaptive Immune Response

#### 6.1.1. Antigen-Presenting Cells in the Epithelium

HPV exclusively infects the epithelium at mucosal sites and the epidermis of cutaneous skin, thereby limiting the expression of viral genes to those cells. Langerhans cells (LCs) are a population of antigen-presenting cells interspersed in stratified epithelium and are thus located in proximity to virally infected cells. LCs in cutaneous epithelium are macrophage lineage derived from foetal liver monocytes and yolk sac macrophages during embryogenesis [117]. Under inflammatory conditions, Gr-1^hi^ monocytes give rise to short-lived LCs in the skin [118]. In mice, oral mucosal LCs are derived from bone marrow-derived pre-DC and monocyte precursors, rather than from foetal liver and yolk sac macrophages [119].

In addition to LCs, the vaginal epithelium also contains dendritic cells described as ‘DC2-like’. These cells are CD11c and CD1c positive, express langerin, and do not contain Birbeck granules [120,121]. We also identified a CD11c positive population in mucosal epithelium in progressing and regressing CIN 2, showing that DC2 cells are also present in cervical epithelium [122]. Liu et al. (2021) described two steady state LC subsets in human inner foreskin: LC1 and LC2 [123]. Bertram et al. later reported that the LC2 subset was DC2 [124]. Liu et al. (2021) also identified two subsets differentiated from steady state LC: activated LC3 (CD83, CCR7^lo^) and migratory LC4 (CCR7^hi^) [123].

LCs express langerin/CD207, a membrane-bound C-type lectin receptor involved in antigen uptake and the formation of Birbeck granules [125,126]. They also express epithelial cell adhesion molecules, including EpCAM/CD326 and E-cadherin [127,128,129,130,131]. Upon the downregulation of E-cadherin on their cell surface, LCs traverse the basement membrane by deploying proteolytic enzymes and mechanical forces, migrating to draining lymph nodes [132,133]. LCs are highly effective antigen-presenting cells in vitro [134]. While there is good evidence that LCs can present antigen via MHC class II and prime CD4 T cells, their role in presentation and cross-presentation of antigen via MHC class I to prime CD8 T cells is less clear.

Given their proximity to infected epithelial cells, it is important to consider whether skin-resident LCs are infected by HPV and whether they are competent to present and cross-present skin-expressed antigens via MHC class I to prime CD8 T cells, or via MHC class II to prime CD4 T cells in vivo. Regarding measles virus (MV), both immature and mature primary lung LCs bind the virus via langerin, but only mature LCs are infected, via CD150 [135]. CD4 T cell responses are activated by both immature and mature LCs following binding. However, while the mature, infected LCs were able to present MV peptides and prime CD8 T cells, they were not able to cross-present inactivated MV or MV-infected apoptotic cells [135]. These findings are consistent with studies using HSV, but contrast with other studies using influenza, where in vitro generated LCs did cross-present the matrix protein and generate CD8 T cell-specific responses [136]. Furthermore, in mice, there is cross presentation of OVA peptides and a response to skin-expressed ovalbumin [137]. We showed that the co-expression of HPV16 E7 and OVA in murine epidermis reduced the number of LCs in the skin and suppressed the CD8 T cell response to OVA. However, LC ablation did not alter either the suppression of the T cell response by E7 or the antigen response to OVA in the absence of E7. This indicates that the CD8 T cell response to OVA expressed in the epidermis occurs in the absence of LCs and that the E7-mediated suppression of that response is independent of LCs [138].

Because the completion of the papillomavirus lifecycle depends on epithelial cell differentiation, productive infection of antigen-presenting cells is widely considered to be unlikely [7]. Moreover, although not directly tested, the enhancer element for the early p97 promoter is only active in keratinocyte-derived cells, not fibroblast-like cells [139]. Although virions can be taken up by antigen-presenting cells [140], RNA transcription is restricted primarily due to the viral E8^E2 repressor protein, which limits expression in non-permissive cells [141]. It therefore seems unlikely that even limited gene expression occurs in antigen-presenting cells. However, there is evidence that papillomavirus-like particles are taken up by monocyte-derived DCs via micropinocytosis and clathrin-mediated uptake, and by monocyte-derived LCs via non-clathrin, non-caveolae-mediated uptake [142]. LCs are not activated (i.e., do not upregulate surface costimulatory molecules, IL-12 expression or migration) following endocytosis of HPV16 VLPs, whereas DCs are [140]. This lack of responsiveness further suggests a significant role for skin DCs rather than LCs in the uptake and presentation of HPV antigens.

For HPV, extracellular antigen release is limited to exposure to viral capsids during infection. However, the intercellular transfer of viral antigens to antigen-presenting cells (APCs) may occur through various mechanisms. APCs acquire intracellular antigens from intact cells via actin-based protrusions known as tunnel nanotubes, which can extend up to 150 µm in length, or via connexin hexamer channels at gap junctions. In hyperplastic tissue (as seen in HPV infection and neoplasia), LC dendrites are reoriented to extend toward the epithelial surface, maintaining contact with cells throughout the epithelial thickness [143].

Dendrite/nanotube transfer is bidirectional between keratinocytes and APCs and can include the transfer of mRNA, which can be expressed by the recipient APC [144]. LCs and DCs can also perform trogocytosis, a process in which peptide–MHC complexes and other membrane-associated antigens are nibbled from neighbouring cells. In the case of LCs, peptide–MHC class II complexes are then transferred to migratory dermal DC2 and double negative DCs, which induce a CD4 T cell response [145]. Cells can also shed extracellular vesicles into the intercellular space, enabling contact-independent antigen transfer [146].

The APCs in the lamina propria are better defined and include dermal CD103+ DC1 (CD141+) and CD1a+ DC2 (CD1c+) subsets, as well as CD123 positive plasmacytoid DC (pDC), which are typically present in low numbers but increase during inflammation. In tissues, DC1 and DC2 are CD11c positive, while pDC are CD11c negative [147]. DC1 cells are highly effective at cross-presenting antigens from keratinocytes and can do so in the absence of LCs [148]. DC2 are efficient at antigen uptake and MHC class II presentation, promoting Th2 responses. DC2 cells also regulate mucosal Th17 cells [149].

Several studies have shown that antigens can be transferred from migratory DCs from the tissues to lymph node resident DCs, which are highly efficient in T cell priming [150,151]. In contrast to conventional DC1 and DC2, pDCs are less involved in antigen presentation but constitutively express TLR7 and TLR9, allowing them to sense viral nucleic acid present in endosomes and respond by secreting IFN-α (reviewed in [152]).

#### 6.1.2. Uptake of HPV Antigens by Local Antigen-Presenting Cells

It is generally believed that APCs primarily function to process antigens and present them to T and B cells in the lymph nodes. In the case of the cervix, the sentinel draining lymph nodes are the left and right obturator fossae, the left and right external iliac, the right common iliac, and the junction of the right internal iliac and obturator fossae [153]. Following antigen presentation in the lymph nodes, antigen-specific lymphocytes are activated and differentiate into effector cells that then migrate back to the tissues to exert their effector functions.

Wang et al. (2015) showed that local immune priming of naïve T cells can occur within mucosal tissues, challenging the view that adaptive immune responses are only induced in the lymph nodes [154]. They identified recirculating naïve CD8 T cells, primarily in the lamina propria, that were stimulated by local CD11c positive APCs clustered in structures termed inducible vaginal lymphoid tissue (IVALT) following intravaginal immunisation in a mouse model [155]. The primed CD69 positive T cells expanded locally and were activated more rapidly than their lymph node counterparts. Importantly, sterile protection against an intravaginal HSV-2 challenge in mice lacking secondary lymphoid organs confirmed the functional relevance of the local T cell response, even though a lower frequency of HSV-2-specific IFN-γ-producing CD3 T cells was generated [156].

### 6.2. The T Cell Response Against HPV

#### 6.2.1. T Cells in the Epithelium

Tissue-resident T cell compartments include effector memory T cells (T_EM_), terminally differentiated effector memory cells (T_ERMA_), and naïve cells. The cutaneous epithelium of an adult human consists of a combination of functionally distinct populations of recirculating (T_RECIRC_) and CD69 positive resident memory T cells (T_RM_), which include both CD4+ and CD8+ subsets. T_RM_ cells can be further characterised into CD103+ and CD103− populations, with CD69+CD103+ T_RM_ cells enriched in epidermis and CD69+CD103− T_RM_ cells more commonly found in the dermis.

The T_RECIRC_ cells are subdivided into CCR7+/L-selectin (+) central memory T cells (T_CM_) and the CCR7+/L-selectin (−) migratory memory T cells (T_MM_). T_RECIRC_ cells are less frequent than the T_RM_ cells, and CD4 T cells dominate over CD8 T cells in the dermis [157]. T_RM_ cells have a limited capacity for proliferation but produce more cytokines than T_RECIRC_ cells, making them more functionally active.

In the ectocervix, most of the CD8+ T cells in the epithelium and stroma are T_RM_ cells, outnumbering CD4 T_RM_ cells. CD103+ T_RM_ cells predominate in the epithelium compared with the stroma [158]. In HSV-2 seropositive individuals, skin-resident CD103+ and CD103− HSV-2-specific CD8 T_RM_ cells are detectable. The CD103hi CD8 T_RM_ cells express granzymes whereas the CD103lo CD8 T_RM_ cells produce IFN-γ [158].

Bystander T cell activation may also occur [159]. For instance, IFN-γ secreting, granzyme B+ CD8 T_RM_ cells not specific to HSV-2 in the genital tract can confer partial protection following viral challenge [160]. Reduced HSV-2 viral loads and clinical symptoms following infection were linked to local recruitment and proliferation of CD8 T cells that were not specific for HSV-2, in response to type I IFN release [160]. This bystander effect suggests that the T cells that infiltrate in inflammation or infection may confer a state of heightened immune surveillance even if they are not specific for the pathogen.

#### 6.2.2. T Cell Effector Function in Viral Infection

Several studies have evaluated peripheral responses to T cells and their correlation with disease regression. In a cohort of HPV18 E6 CD4+ T cell responders, strong Th responses to E7 were associated with an HPV18 negative status, suggesting that the response was associated with clearance [161]. CD4+ T cell responses to HPV16 E6 peptides correlated with disease regression, whereas E7-specific responses were increased but not statistically significant [162]. Our group showed that Th1 responses to HPV16 E2 peptides were associated with the regression of low-grade lesions, with no requirement for E2-specific CD8 T cell responses to a selection of E2 peptides [163]. A longitudinal study confirmed that IFN-γ ELISpot responses to HPV16 E2 peptides were significantly associated with lesion regression, whereas non-responders to E2 or E7 tended to progress [164].

CD4+ T cells, characterised by a cytotoxic gene signature, are detected in peripheral blood primarily in chronic infections such as Epstein–Barr virus (EBV), CMV, and HIV. These cells target MHC class II-expressing cells, including antigen-presenting cells, B cells, and also infected or transformed cells with upregulated MHC class II. The EBV-specific CD4+ cytotoxic T cells in peripheral blood are highly differentiated, antigen experienced, memory T cells with decreased expression of the costimulatory molecules CD28 and CD27 and increased expression of intracellular cytotoxic granules and perforin [165]. These cells are effective killers of EBV-transformed B cells in vitro.

In the skin, increased numbers of CD4+/perforin+ CD69+ T_RM_ cells predominate in the dermis of older individuals (16–28 years old c.f. 53–74 years old) and are more common than CD8+/perforin+ cells [166]. In the case of human CMV, around 20% of the perforin positive skin-resident CD4 T cells are specific for an HLA-DR7 restricted human CMV-gB peptide and were cytotoxic for human CMV+ fibroblasts in the dermis [166]. The transcription factor B lymphocyte-induced maturation protein (Blimp-1) upregulates the production of granzyme B and development of cytotoxic activity by CD4+ T cells. Hobit, which is homologous to Blimp-1 in T cells, is specifically upregulated in T_RM_ cells [167] and is associated with the cytotoxic potential in CD4 T cells [168].

MHC class II expression on cells is required for them to be targeted by cytotoxic CD4 T cells. While some MHC class II positive cells can be detected in normal cervical epithelium, CIN 1 lesions have increased numbers of MHC class II positive cells and MHC class II is upregulated with increased viral load [169]. This suggests that infected keratinocytes may be targeted by cytotoxic CD4+ T cells. Although not directly studied in HPV-infected tissues, there is a substantial increase in the number of CD4+/NKG2D+ cells in the peripheral blood of individuals with CIN 1 compared to normal controls [170]. These cells are perforin positive and typically CD28 negative, which is consistent with the cytotoxic phenotype.

#### 6.2.3. Local T Cell Responses and Disease Regression

In the canine oral papillomavirus model, immune regression correlates with an infiltration of CD4 and CD8 T cells, with CD4 T cells being most abundant [171]. Initially, a CD4 infiltration into inflammatory foci in the lamina propria is seen. At the peak of the response, the infiltration into the lesion included CD4 and CD8 T cells, in addition to CD1a and c and CD11a, b, and c positive cells. The authors propose a primary and initial role for CD4 T cells in regression. They suggest that the subsequent infiltration of antigen-presenting cells follows a loss of epithelial integrity, and that this infiltration may be in response to tissue necrosis in the regressing wart [171].

A definitive role for both CD4 and CD8 T cells in the control of cutaneous infection is evidenced in mouse papillomavirus (MmuPV1). Depletion of either CD4 or CD8 in C57Bl/6 mice is insufficient for productive infection, whereas CD3 depletion renders mice competent for infection [155]. Surprisingly, type 1 IFN knockout mice were also not competent for MmuPV1 infection, despite the important role for type 1 IFNs in the innate response to virus infection [155].

While CD4 and CD8 T cells are detected in biopsies of HPV lesions, CD4 T cells are more frequent. Coleman et al. (1994) carried out a cross-sectional analysis of 14 HPV-positive women with regressing genital warts and compared them with 14 HPV-positive women with persistent warts [172]. There was a predominant infiltration of T cells concentrated beneath the lesion, as well as macrophages. Few B cells or large granular cells were seen, and the numbers of LCs present did not differ between regressing and persistent lesions. While most of the stromal T cells were CD4+ in persistent lesions, CD4+ and CD8+ T cells were detected in the stroma and epithelium of regressing tissues [172].

In a study of regressing versus progressing CIN 1, the ratio of CD4:CD8 positive cells in the stroma was increased; however, this was not statistically significant perhaps due to a small sample size (regressing CIN 1: n = 9; progressing CIN 1: n = 5) [173]. In the same study, the general trend was for CD8+ T cells in the epithelium and stroma to be found in higher numbers with higher grade CIN, and for CD4 T cells to be found in lower numbers, suggesting that CD8 T cells are less effective in controlling disease than CD4 T cells [173]. This may be contributed to by the downregulation of MHC class I on the target cells [174], rendering the CD8 T cells less effective in cytotoxic killing.

A more detailed analysis of CD4 subsets was included in a study of persistent and regressing versus progressing CIN 2 [122]. As is consistent with other studies, persistent and regressing disease was associated with significantly more stromal CD4 and CD8 T cells and CD11c+ cells compared with progressive disease. Of the T cell subsets, CD4 T cells were more frequent than CD8 T cells. Cells positive for the transcription factor Tbet, which is expressed by Th1 cells, were increased in the lesional tissue of the persistence/regression group, suggesting that infection and disease progression is not controlled when their numbers are reduced. In contrast, GATA3 positive and IL-17 positive cells were not significantly different in number between the two groups [122]. Furthermore, the CD8 T cell granzyme positivity did not differ between the persistence/regression and progression groups. Most of the CD8 T cells were granzyme negative. Granzyme was expressed on 1% of the CD8 T cells in the epidermis and around 7% of the CD8 T cells in the dermis. The association between increased numbers of CD4 T cells and the regression of CIN 2 has been corroborated by others [175].

### 6.3. The Antibody Response to HPV Infection

There is a highly effective prophylactic vaccine for high-risk HPV that generates systemic antibodies against L1 protein, and there is a correlation between vaccine-induced antibodies in the serum and antibodies in the cervicovaginal secretions, indicating that systemic antibodies transudate through the vessels into the epithelium and the genital tract. In contrast, in natural infection viral antigens are exposed to the mucosal compartment of the immune system. The activation of a mucosal response can stimulate systemic antibodies and secretion of antibodies at other mucosal sites [176]. Systemic antibodies to L1 generated in response to natural infection with HPV16 are detected in only around 50% of individuals.

#### 6.3.1. B Cells in Tissues

Following antigen uptake, antigen-presenting cells are required to migrate from the tissue to tertiary lymphoid structures such as lymphoid follicles and germinal centres that form locally in the tissue, or to the local lymph nodes to activate an adaptive immune response. Lymphoid aggregates of CD19/CD20+ B cells surrounded by CD4+ and CD8+ T cells have been detected in normal cervical tissues [177]. While this has not been looked at specifically in the context of HPV infection, lymphoid aggregates that were defined as a core of CD20+ B cells interspersed with CD68+ macrophages and CD4 and CD8+ T cells and an absence of tingible-body macrophages (defined phenotypically by staining and the phagocytosed apoptotic cells contained within them) are increased in HSIL (57% of positive cases c.f. 9.5% in normal cervix) [178]. Interestingly, a distinct CD8+ T cell dominant type of lymphoid aggregate is formed in HIV+ HSIL in 50% of cases (c.f. 0% of HIV− HSIL). In addition, dense cellular accumulations of B cells with interspersed CD4 T cells and macrophages (including tingible-body macrophages), surrounded by CD4 and CD8 T cells, consistent with germinal centres that typically form in secondary lymphoid structures, were identified and reported to be increased in HSIL (33% of positive cases c.f. 4.7% in the normal cervix) [178]. Germinal centre formation in the cervix is not unique to HPV and HIV and is also associated with *Chlamydia trachomatis* infection and cervicitis [179,180]. While the prognostic importance of tertiary lymphoid structures has not been explored in HPV infection, tertiary lymphoid structures (TLSs) correlate with increased patient survival in a range of human tumours. In the context of HPV, germinal centre tumour-infiltrating B cells are increased in HPV-positive compared with HPV negative head and neck cancers and correlate with better outcomes [181]. In cervical cancer, TLSs containing tumours are enriched in immune cells, with more antigen-presenting cells co-located with CD8+ T cells and more immune checkpoint positive T cells [182]. The presence of TLSs may therefore predict responsiveness to specific therapies such as checkpoint inhibitors.

B cells have other functions aside from the generation of antibodies, including antigen presentation and T cell activation, proinflammatory cytokine release, and immune regulation through the secretion of IL-10 [183]. B cells from non-small cell lung cancer present antigen to T cells in vitro and can shape the T cell response [184]. If activated, the B cells are associated with a Th1 phenotype, whereas exhausted B cells are associated with the development of Tregs [184].

It is now feasible to determine the specificity of B cells from tissues through single cell sequencing and the expression of cloned IgG heavy and light chains into 293 cells. Although this has not yet been described for productive HPV infection, in HPV-positive head and neck cancer, a localised response by activated B cells was directed at E2, E6, and E7 (the only HPV antigens tested), with E2 responses dominating [185]. The functional significance of antibodies to viral intracellular proteins is unclear. While tumour-specific antibodies can enhance the anti-tumour immune response through the activation of NK-mediated antibody-dependent cellular cytotoxicity (ADCC), this is restricted to cell surface tumour-specific antigens because intracellular antigens are not typically bound by antibodies. However, antibody-bound viral capsid proteins can be internalised by cells and are bound via Fc by cytosolic TRIM21, which is upregulated in the presence of IFN. Following binding, the complex is rapidly targeted for degradation via the proteosome in a process known as antibody-dependent intracellular neutralisation (ADIN) [186].

#### 6.3.2. Antibodies in HPV Infection

It is important to consider antibody responses to HPV in the context of viral antigen expression and its location during the virus lifecycle. While E4 is abundantly expressed, as are the late proteins, E2 is upregulated in the upper layers of the epidermis and E6 and E7 are not considered to be expressed at high levels in HPV infection. Effectively all early viral proteins are intracellular, and virions are shed in squames, limiting the exposure of the immune system to viral antigens. However, E1^E4 induces weakening of the cornified cell envelop of the virion-laden cells by disrupting the intermediate filament cytoskeleton to enable virions to be released from squames to infect other cells [187]. Antigen-presenting cells are therefore exposed to extracellular L1 in its native configuration, accounting for the detection of neutralising antibodies following natural infection. The neutralising antibody response in some people may relate to viral load as the lesion progresses, or other factors such as inflammation or co-infection, that are sufficient to counter the suppressive effects of HPV on the immune response.

Antibodies to HPV are maternally transmitted and acquired early in life. The vertical transmission of HPV6 is of particular importance because of the association with juvenile-onset laryngeal papillomas. A comprehensive analysis of maternal and neonatal antibodies was completed on 272 children and their mothers with vertically acquired HPV6 infections [188]. There was a significant correlation between maternal and neonatal antibodies to HPV6 proteins. L1 seroconversion occurred at around 12 months and seroconversion to the early proteins occurred by around 24 months, following the decline of maternal antibodies in the neonates. Of the individual viral antigens, antibodies to L1 were most readily detected, followed by E2 then E4 and E6, with the lowest levels detected against E7. The gradual increase in antibodies to early proteins following acquisition of vertically transmitted HPV6 in infants contrasts with the more pronounced L1 antibody response in these individuals and suggests that the early protein antibodies develop in response to the gradual release of these antigens over time.

Several studies have examined the response to the viral antigens in cervicovaginal secretions, with IgA or IgG responses to a range of viral antigens, including L1 virus-like particles, synthetic peptides, or protein for L1, L2, E2, E4, or E7 being measured. Antibodies to L1, L2, E2, E4, or E7 have been detected in the cervicovaginal secretions of some, but not all individuals, with condylomas or LSIL (reviewed in [189]).

#### 6.3.3. Antibody Function in HPV Infection

While antibodies to early proteins have no functional significance because they are not able to access these intracellular viral antigens, neutralising antibodies against L1 bind to the viral capsid, preventing infection. HPV virions are made up of L1 and L2 containing capsids. However, L2 is only exposed following capsid binding to heparan sulphate proteoglycans on the basement membrane, and it is then proteolytically cleaved by furin (reviewed in Schiller et al. 2010) [190]. Therefore, L1 is the primary target for virus neutralisation. The infection process for HPV is complex and relatively slow (12–24 h), which further increases the vulnerability of HPV to antibody neutralisation [191]. Carter et al. (2000) carried out a comprehensive longitudinal analysis of capsid antibody responses to HPV16, 18, and 6 in HPV DNA detectable infections in 18–20-year-old women (n = 30–42 women, depending on HPV type) [192]. They found that the median time to seroconversion was around 12 months, irrespective of HPV type, and while 85–90% of HPV 6 and 16 individuals had seroconverted by 18 months, only 50% of HPV18 positive individuals seroconverted.

#### 6.3.4. Antibodies to HPV and Disease Regression

In canine oral papillomavirus infection, seroconversion to L1 antigen follows the infiltration of cells into the site of infection [171]. Although L1 antigen was detectable in the lesion at weeks 7 and 8 post-infection, virus-specific serum antibodies to L1 were not detected at that time. Regression occurred between weeks 8 and 10; however, antibodies were not measured at weeks 9 and 10 but were presumably increasing because they were readily detectable at week 11. Therefore, seroconversion occurs after infiltration of immune cells in the lesion, but the direct association between seroconversion and lesion regression is unclear.

The relationship between naturally acquired antibodies to L1 and disease regression or progression of CIN is also not clear. In HPV L1 antigen positive individuals with cervical dysplasia, 26% of women were HPV16 L1 antibody positive compared with 4% of the control, the L1 antigen negative group. At 66 months follow up, 8.7% of cases retained L1 antigen and were HPV16 L1 antibody positive [193]. In another study, 69% of women who cleared HPV16 were positive for HPV16 VLP-specific IgG compared with 100% of women with progressive HPV16 disease [194]. Ochi et al. (2012) also reported an association between persistent disease and progression in women with HPV16-specific neutralising antibodies [195]. L1 antibody detection does not have prognostic utility clinically for HPV-associated disease.

## 7. The Tissue Microenvironment in HPV-Positive High-Grade Disease

Some level of integration of viral genomes and increased expression of E6 and E7 following the deregulation of the control of the p97 promoter is characteristic of high-grade disease. Changes to the epithelial cells are primarily a result of increased E6 and E7 expression. These changes include an increased nuclear to cytoplasmic ratio in cells, de-differentiation of the epithelium, and a resultant increase in cells in the transit amplifying layer of the skin.

Molecular and cellular changes in the tissue microenvironment in premalignant lesions in the absence of HPV are demonstrated in the lung, where high-grade precancer is associated with the upregulation of inhibitory molecules including IDO1, PD-L1, CTLA4, TIGIT, and TIM3 and with the expression of suppressive interleukins, including IL6 and IL10. There is also co-regulation of activated T cells, memory B cells, follicular T helper cells, DCs, and NK cells [196].

In the case of HPV-positive HSIL, HPV-mediated direct effects on the local immune microenvironment may contribute to progression in cervical neoplasia, in addition to indirect effects that result from the epithelial changes to cells driven by the integration of the viral genome and upregulation of E6 and E7 (increased proliferation, thickening of the epithelium). The tissue microenvironment therefore reflects both the direct effects of HPV and indirect effects on the cells and their altered differentiation in the epithelium.

At the cellular level, the tissue microenvironment in high-grade cervical disease is characterised by increased cells of the myeloid lineage, regulatory T cells, including FoxP3 positive cells, and reduced numbers of CD4 and CD8 T effector cells and B cells. Clusters of MAIT cells are present, as are granulysin-positive NK cells (Figure 1C).

At the molecular level, Ovestad et al. (2022) carried out a comparison of the expression of 398 immune response genes between HSIL/AIS and normal cervical biopsies and identified 27 differentially expressed genes between normal and CIN 3 lesions [197].

They identified decreased expression of the NK cell marker *NCAM1* (CD56), which was attributed to a loss of CD56 positive cells with strong *NCAM1* expression, which are more frequent in normal biopsies. They also found that *CX3CL1*, which normally promotes NK cell activation, was downregulated. *CD160* and *IL18* were also downregulated.CD160 and IL-18 proteins are required for NK cell-mediated IFNγ production.

A range of other inflammatory chemokines were also downregulated, including *CCL5* and *CXCL11*, suggesting a dampening of the adaptive immune response [197]. Genes associated with proliferation, increased plasticity, and invasion were increased in CIN 3 lesions.

### 7.1. TLRs Are Regulated in High-Grade Disease

HPV persistence and disease progression are reflected in changes in innate molecule expression, including TLRs. HPV deregulation of the keratinocyte-mediated inflammatory response may contribute to virus persistence [42]. Type 1 IFN can directly induce TLRs 1, 3, 5, and 7 [198]. DeCarlo (2012) showed that these TLRs, while upregulated in the stroma, are not upregulated in HPV-positive HSIL [35]. This lack of expression in the epithelium may contribute to the persistence and ultimately the progression of HPV-positive lesions. In contrast, Hasimu et al. (2011) found a positive correlation between the expression of TLR4, TLR7, and TLR9 and the progression of CIN [199].

### 7.2. Myeloid Cells Regulate the Tissue Microenvironment in High-Grade Disease

There is data to suggest that progression to high-grade disease is associated with a dominance of regulatory immune cells over effector cell subsets. For example, vulval dysplasia caused by HPV is characterised by an increase in intraepithelial and stromal mature CD14+ macrophages and an inverse association with CD8+TIM+ T cells [200]. Higher numbers of intraepithelial myeloid cells also correlate with the presence of intraepithelial regulatory T cells, consisting of CD4+FoxP3+, CD3+PD1+FoxP3+, CD4+TIM3+ T cells [201].

CCL22 is a chemoattractant for a range of immune cells, including T-regs, and there is a positive correlation between CCL22 positive cells and FoxP3 positive cells in cervical dysplasia tissues. Furthermore, CD68 positive and FoxP3 positive cells were co-located in these tissues. The majority of CD68 positive cells were also CCL22 positive and M2 polarised, as assessed by PPARγ staining. The authors propose that CD68 positive macrophages secrete CCL22 and that this facilitates the recruitment of FoxP3 positive cells to the tissue [202].

Tissue macrophages are categorised into tumour suppressive type 1 macrophages (M1-like), which produce IL-12 and TNFα, and tumour promoting type 2 macrophages (M2-like), which produce anti-inflammatory cytokines. Higher numbers of M2 over M1 macrophages in exfoliated cell samples from the cervix is associated with increased risk of progression in HPV infection [18].

Spatially, ‘immune surveillance’ clusters containing myeloid cells were identified by single cell sequencing of HSIL biopsies [15]. *TNFRSF10B* (expressing DR5/TRAIL-R2) myeloid cells are clustered together with *TNFSF10* (expressing TRAIL) positive HPV-related HSIL epithelial cells in HSIL foci. *TNFRSF10B* encodes theTNF-receptor superfamily member TRAIL-R2 that is activated by apoptosis-inducing ligands such as TRAIL. The clusters in HSIL were associated with apoptosis of the abnormal cells [15].

In addition to the myeloid immune surveillance clusters, immune inhibitory CD46+ CD8+ MAIT cells clustering with *JAG1*+ HPV-related HSIL epithelial cells were also identified [15]. The JAG1-CD46 interaction drives differentiation of CD4+ cells to Tregs in mice [203]. However, the JAG1-CD46 interaction is also critical for human Th1 immunity [204].

In contrast to the tissue, the exfoliated cells of the cervix have reduced representation of myeloid cells in HSIL compared with LSIL. Associated with this was the increased representation of genes (including *SPP1*, *GPNMB*, and *CD163*) associated with lipid-associated macrophages (LAM) [18]. SPP1 (osteopontin) is an intracellular protein and GPNMB is a ligand for syndecan-4 on T cells that inhibits their activation and proliferation [205]. CD163 is a scavenge receptor for the haemoglobin–haptoglobin complex, which drives M2 phenotype cytokine expression (including IL-6 and IL-10) following binding [206,207]. The functional significance of these LAM cells is currently not known.

### 7.3. Lymphocyte Subsets and Numbers in High-Grade Disease

A comparison of HPV-positive high-grade squamous intraepithelial lesions (HSILs) compared with HPV-positive cytology negative samples identified by single cell sequencing showed a decreased proportion of CD4 positive and CD8 positive T_EM_ cells in tissue biopsies [15]. Zhang et al. (2023) carried out single cell sequencing analysis of T cells in normal cervix, HSIL, and cancer, and proposed a reduction in Th cells and an increase in Treg in HSIL, which progressed to an increase in activation-coupled exhausted CD8 T cells and reduced cytotoxic CD8 T cells in microinvasive cervical cancer [208]. In another report, reduced CD8 T_EM_ and T_RM_ cells and increased granulysin positive NK cells were identified in HSIL when compared with HPV-positive normal cervical biopsies [209]. Reduced numbers of T cells in exfoliated cell samples from the cervix is also associated with increased risk of progression in HPV infection [18]. Additionally, Saito et al. (2022) showed that CIN 2 lesions that went on to progress in a 6–18 month follow up had significantly lower numbers of CD4 and CD8 T cells and CD11c positive DCs in the lamina propria compared with CIN 2 lesions that went on to persist or regress [122]. The reduced numbers of effector T cells in the lesion may be contributed to by the dysregulated expression of vascular adhesion molecules. MAdCAM-1 expression on endothelial cells is downregulated in CIN 3. MAdCAM-1 is the ligand for the homing integrin expressed on cervical T cells, a4b7, thereby precluding egress of T cells into the dysplastic epithelium [210].

Other changes in T cell populations identified by a single cell sequencing comparison of HPV-positive HSIL with cytology negative samples included an increased proportion of naïve CD4 T cells (*CCR7*, *SELL*, *LEF1*) and ‘Th1-like’ cells (*CXCL13*, *PDCD1*, *IFNG*), and a decreased representation of Th17 (*IL17A*, *CCR6*, *CTSH*) cells in tissue biopsies [15]. In contrast to these data, Saito et al. (2022) [122] showed that low numbers of T-bet positive cells in the CIN 2 lesion were significantly associated with disease progression. Furthermore, although Guo et al. (2023) [15] identified a decreased representation of Th17 cells; there is significantly more IL-17 secreted from CIN 3 than from HPV-positive cytology normal or from CIN 1 cervical tissue homogenates [211].

There is also reduced numbers of B cells in the exfoliated cells of the cervix in HSIL compared with LSIL. Plasma cells (CD138+) are readily detected in HSIL and are distributed throughout the lamina propria where they do not closely aggregate with T cells [178]. Plasma cells were found in CIN 2 lesions but were not significantly associated with subsequent disease regression or progression [122].

### 7.4. Predicting Disease Progression in High-Grade Disease

While there are several studies that have looked at differences between low-grade and high-grade cervical neoplasia, only a few studies have looked at predictors of disease progression within a grade of lesion. In the study by Saito et al. (2022) the tissue microenvironment of CIN 2 was characterised in groups separated based on subsequent disease outcomes (progression versus persistence/regression). There was reduced numbers of Th1 cells in the lesion in the group that went on to progress and increased numbers of cells expressing immune regulatory molecules in the lesion, including Blimp-1, FoxP3, and HMGB1, relative to the persister/regressor group [122]. Disease progression is also associated with low CD4 and CD8 T cell and CD11c+ cell numbers in the lamina propria, compared to persistence/regression. Blimp-1 play a direct role in disease progression, or it may function in association with the immune cells activated in response to HPV antigens [122].

## 8. Lessons Learnt from Therapeutic Vaccination for HPV-Associated Cervical Neoplasia

The current prophylactic vaccines available against HPV do not show any significant therapeutic effect on pre-existing infections or high-grade HPV-associated CIN, which is not surprising considering that antibodies have limited exposure of viral capsids during active infection and at best could only limit the spread of infection on the surface of the epithelium [212]. There remains a clinical need for immunotherapeutic interventions that treat existing disease and prevent disease recurrence.

A therapeutic vaccine for HPV-associated neoplasia must be able to induce an effective antiviral response by initiating an immune response that clears the infection, resulting in lesion regression. It should trigger an innate immune response, generate immune memory, and modulate or reverse the immunosuppressive tissue microenvironment at the lesion site. Both CD4 and CD8 T cells are essential for the control of papillomavirus infection. Because E6 and E7 expression is increased following the integration of the viral genome in high-grade neoplasia and because these proteins have a vital role in the initiation and progression of malignancy, they have been most frequently targeted for the development of therapeutic vaccines.

Various approaches, including peptide-, protein- and cell-based, bacterial- and viral-vectored, and DNA/RNA and combinations thereof have been employed for the development of HPV therapeutic vaccines. However, no therapeutic vaccine has been sufficiently efficacious for the treatment of HPV-positive pre-malignant lesions to be routinely used clinically.

### 8.1. T Cells Expand in the Cervix Following Systemic Vaccination

One consideration for an immunotherapeutic vaccine is whether systemic immunisation at a non-mucosal site can result in T cells at the lesion. Intramuscular vaccination using a heterologous HPV16 E7 DNA and HPV16 and 18 E6 and E7 vaccinia vector-based boost elicited antigen-specific IFN-γ secreting cells in the peripheral blood in around half of the vaccinated participants. The cervical stroma and dysplastic epithelium showed an infiltration of CD8+ T cells, including Tbet positive cells, and there was evidence of apoptosis at the site. Furthermore, TLSs were present as well as a molecular signature with expression of genes that suggest immune cell activation (*CXCR3*), Th1 polarisation and effector function (*Tbet* and *IFN-β*) [213]. This study does demonstrate that intramuscular vaccination can result in clonal expansion of appropriate effector T cells to the cervix.

### 8.2. Not All Antigens Are Equally Effective

The combined use of E6 and E7 is more effective than E7 alone. A DNA vaccine targeting HPV 16 E7 that had been ‘detoxified’ by mutation and that was fused to HSP70 and tested in women with CIN 2 vaccination elicited a weak HPV-specific T cell response in peripheral blood and only a minor effect on histological regression (33% in the vaccinated group versus 20% in the control group) [214]. Trimble et al. (2015) assessed the immune response generated by VGX-3100, a DNA vaccine designed to target HPV 16 and 18 E6 and E7 delivered by electroporation. Participants with CIN 2 and 3 who received the vaccine and demonstrated histopathological regression showed increased CD8 T cells in the stroma compared with those that did not regress (49.5% in the vaccinated group versus 30.6% in the placebo group) [215]. They also demonstrated a greater magnitude of an ELISpot response to E6 in the periphery, whereas the magnitude of the E7 response did not correlate with regression. The differences in antigens targeted and impact on vaccine efficacy indicate the importance of the inclusion of multiple antigenic targets in a therapeutic vaccine for HPV-associated CIN. The inclusion of other HPV antigens in therapeutic vaccines also has merit, as it increases the peptide pool to which T cells can respond.

### 8.3. The Magnitude of the T Cell Response Correlates with Disease Regression

In addition to the study by Trimble, who demonstrated that the magnitude of the E6 response in the periphery correlated with regression, this has also been shown with long-peptide vaccines. These vaccines have shown some efficacy, and the peripheral T cell response to the vaccine antigens correlates with disease regression. For example, ISA101 consists of two mixtures of in total 13 synthetic long peptides (25–35 amino acids in length; overlapping by 10–18 amino acids) covering the entire amino acid sequence of HPV16 E6 and E7 oncoproteins. Subcutaneous vaccination with ISA101 induced partial or complete histologic regression of lesions in more than 50% of patients with HPV16 positive associated high-grade vulvar intraepithelial neoplasia (VIN) [216]. A total of 85% of participants displayed an HPV-specific IFN-γ-associated T cell response in the peripheral blood, and stronger T cell responses were associated with disease regression.

### 8.4. TLR Agonists May Be Effective in Activating T Cell Responses to Endogenous or Exogenous HPV Antigens

Because of their wide-ranging impact upon both innate and adaptive immunity, TLRs and their signalling pathways have emerged as attractive therapeutic targets that could function as immunological adjuvants [217]. Imiquimod is a TLR7 and 8 agonist that activates the innate immune response by stimulating dendritic cells followed by a cellular immune response [218]. This agnostic activity leads to activation of NF-κB and induction of proinflammatory cytokines that stimulates Th1-cellular immune response. In a clinical trial of E6 and E7 long peptides and VIN, one arm included the topical application of Imiquimod to the vaccination site [216]. This did not impact the efficacy of the vaccine; however, it would have been interesting to determine if topical application of Imiquimod to the lesion rather than the site of immunisation increased the regression frequency in vaccinated women, in consideration to reports of upregulated TLR7 expression in progression and regression [39,199].

*Candida* antigen activates TLR2 and 4. PepCan is a peptide-based vaccine covering HPV 16 E6 protein and Candin, which is an extract derived from *C. albicans* [219]. In a study carried out by Nakagawa et al. (2005), the effectiveness of PepCan and *Candida* were examined. *Candida* was found to be more effective in regressing CIN 2 and 3 compared to PepCan, with HPV-specific T cell responses detected in both groups, suggesting that the inclusion of the E6 peptide may have had some inhibitory effects. Robust changes in the cytokine data in *Candida* group compared to the PepCan group demonstrate that the adjuvant effect may be more significant than the immune stimulation by HPV antigens for therapeutic vaccines [44].

### 8.5. Targeting Druggable Inhibitory Molecules Such as IDO1 in Conjunction with Vaccination May Enhance Vaccine Efficacy

We and others have shown that indoleamine 2,3-dioxygenase 1 (IDO1) is upregulated by HPV16 and is expressed in HPV16 positive lesions [220,221,222,223]. IDO1 is a rate limiting intracellular cytosolic enzyme that catalyses tryptophan to kynurenine. It is induced by IFN-γ, prostaglandin E2, TNFα, TGFβ, and other proinflammatory signals and is expressed by tumour, endothelial and dendritic cells, and macrophages within the tumour microenvironment [224,225]. IDO1 has an immunosuppressive role in tissues [226,227]. High levels of IDO1 in tumours inhibit anti-tumour T cells and is associated with poor prognosis [228,229].

Several inhibitors of IDO1 have been developed. For example, Epacadostat is a potent and highly selective IDO1 enzyme inhibitor which enhances proliferation of effector T cells and NK cells, increases activation of CD86 on dendritic cells, decreases apoptosis, and downregulates Tregs, changing the tissue microenvironment from an immunosuppressive state to a state of productive immune response [230]. Another inhibitor, Indoximod, relieves the inhibitory effects of IDO1-mediated tryptophan deprivation on mTOR signals needed in T cells for anti-tumour activity [227]. Administering inhibitors such as these may increase the efficacy of immunotherapeutic vaccines and trigger T cell-mediated regression by overcoming what is an otherwise immunosuppressive tissue microenvironment.

## 9. Conclusions and Future Directions

It has been around 45 years since Harold zur Hausen and his team established the link between high-risk HPV types and cervical cancer. Today, an effective prophylactic vaccine is in use and HPV testing has replaced cytology as the primary screening tool in some countries. Although the development of a therapeutic vaccine for HPV-positive individuals with cervical neoplasia has been considered important and researched since the 1990s, an effective version has yet to be developed.

HPV infection is protracted compared to many other viral infections. While this review primarily focuses on cervical HPV infections due to their extensive characterisation, many of the findings are likely to be generalisable to HPV infection at other anatomical sites. In cervical disease, high-grade lesions have a higher probability of progression compared to low-grade lesions, while the reverse is true for regression. Persistent infection may be due to a lack of a response to viral antigens, functional unresponsiveness, or partial immune control that is insufficient to trigger immune-mediated regression. Regression is immune-mediated, but the trigger for regression is currently unknown.

The tissue microenvironment of HPV-positive, cytologically normal lesions includes cell populations whose functional significance remains unclear, such as Th17 cells and CD8+ T_ERMA_ cells. Additionally, MAIT cell numbers are increased, although their functional role is also not well understood. This early-stage infection may be facilitated by limited viral antigen availability and virus-mediated avoidance of innate sensing, resulting in immune hypo-responsiveness. The antigen specificity of T cells identified in formalin-fixed, paraffin-embedded biopsy tissues cannot be readily determined, representing a significant gap in understanding of the function of T cell subset functions in HPV infection and disease.

Around 90% of cervical neoplasia cases eventually regress spontaneously through immune-mediated mechanisms. This regression requires both CD4+ and CD8+ T cells specific to viral antigens. However, the immune trigger that shifts the persistently infected state to regression remains unknown. There is also limited understanding of how HPV antigens are taken up and presented locally and in the draining lymph nodes. The relative importance of locally activated T cells versus those in the draining lymph nodes is also unclear.

High-grade lesions exhibit features associated with a higher likelihood of disease progression, including a predominance of suppressor cells at the infection site. This is partly due to increased expression of the immune-suppressive viral E6 and E7 genes and, as observed in premalignant lung disease and the K14 E7 mouse model, the resulting epithelial hyperplasia. Although there are many viral immune evasion mechanisms that have been identified in vitro, their relevance in HPV infection in human disease is less well understood. In the context of immunotherapeutic vaccines, it is also crucial to determine whether there is an immunological ‘point of no return’ in disease progression beyond which regression is no longer possible. However, this remains poorly understood. More detailed, longitudinal analyses of the immune landscape within the lesional microenvironment are needed to better understand the role of the immune response in persistence, regression, and progression.

Single cell sequencing offers an unbiased assessment of the tissue microenvironment and has enhanced our understanding of cervical HPV disease. However, it is costly, and only a limited number of samples have been analysed to date, which may limit the generalisability of the findings. Additionally, single cell sequencing does not provide spatial data and samples may include non-lesional cells. Nevertheless, sample sizes are expected to increase over time, especially if the cost of this technology decreases. Advances in the resolution of single cell spatial imaging will further improve the characterisation of the cell subsets and their likely functions in a spatial context in HPV lesions.

In vitro systems used to study HPV do not fully capture the immunological complexity of the tissue microenvironment. While organotypic raft cultures support differentiation and completion of the viral lifecycle, immune cells and the stroma are not adequately represented. Human tissue explants are difficult to obtain and cannot be maintained viably over the long term. Mouse models using MmuPV1 allow for the evaluation of immune responses, leveraging the extensive array of available reagents and knockout mice. However, MmuPV1 is considered more closely aligned to the beta papillomaviruses that infect cutaneous epithelium than the alpha papillomaviruses that infect mucosal sites such as the cervix. However, MmuPV1 does infect mucosal sites and continues to provide valuable insights into the interaction between papillomavirus and the immune system.

The utilisation of an effective immunotherapeutic vaccine for HPV-associated cervical neoplasia will enhance treatment success and reduce the risk of disease recurrence. An effective immunotherapy will generate HPV-specific CD4+ and CD8+ effector T cells at the site of infection and will overcome local suppression or inhibition of effector T cell function. Evidence suggests that vaccination strategies generate HPV-specific CD4+ and CD8+ T cells and that at least some of them home to the infection site. However, these vaccines have typically provided only incremental improvements in regression rates over the background of spontaneous regression in individuals with high-grade disease. It remains to be determined whether sufficient numbers of antigen-specific T cells are in the lesion, and whether these cells are functional and not suppressed locally. Identifying these barriers will aid the development of an effective therapeutic vaccine.

Combining immune-modifying strategies with vaccination, such as the topical application of TLR agonists or the systemic administration of immune modifying drugs, may enhance vaccine efficacy. Although some evidence supports TLR expression in tissues, results vary between studies, and the functionality of these receptors remains unclear. Improved antibody validation and standardisation for tissue staining are needed to improve consistency across studies. Validation data should be reported, including staining on known positive tissues where available.

In summary, significant progress has been made in understanding the epithelial response to HPV infection, knowledge that is critical for developing effective immunotherapies for HPV-positive cervical neoplasia. However, key gaps must be addressed to increase the efficacy of immunotherapeutic vaccines to a level that surpasses that of the current treatments for high-grade cervical neoplasia.

## Data Availability

No new data were created or analysed in this study. Data sharing is not applicable to this article.

## Abbreviations

The following abbreviations are used in this manuscript:

ADCC	Antibody-dependent cell cytotoxicity
ADIN	Antibody-dependent intracellular neutralisation
AIS	Adenocarcinoma in situ
APCs	Antigen-presenting cells
Blimp 1	B lymphocyte-induced maturation protein
CCL2	C-C motif ligand 2
CD	Cluster of differentiation
cGAMP	Cyclic GMP-AMP
cGAS	Cyclic GMP-AMP synthase
CIN	Cervical intraepithelial neoplasia
CMV	Cytomegalovirus
CTLA4	Cytotoxic T-lymphocyte associated protein 4
DCs	Dendritic cells
EBV	Epstein–Barr virus
FoxP3	Forkhead box P3
GM-CSF	Granulocyte-macrophage colony-stimulating factor
GPNMB	Glycoprotein non-metastatic melanoma protein B (also known as osteoactivin)
HIV	Human immunodeficiency virus
HLA	Human leukocyte antigen
HMGB1	High mobility group box 1 protein
HNSCC	Head and neck squamous cell carcinoma
HPV	Human papillomavirus
HSIL	High-grade squamous intraepithelial lesion
HSV	Herpes Simplex Virus
IDO 1	Indoleamine 2,3-dioxygenase 1
IFI16	IFN-γ-inducible protein 16
IFIT1	ISG IFN-induced protein with Tetratricopeptide Repeats 1
IFN	Interferon
IL-1β	Interleukin 1-beta
iNKT	Invariant NKT
IRF	IFN regulatory factor
ISGs	IFN-stimulated genes
ISREs	IFN-stimulated response elements
IVALT	Inducible vaginal lymphoid tissue
JAK/STAT	Janus Kinase/Signal Transducer and Activator of Transcription
LAIR-1	Leukocyte-associated Ig-like receptor-1
LCs	Langerhans cells
LSIL	Low-grade squamous intraepithelial lesion
MAdCAM-1	Mucosal addressin cell adhesion molecule 1
MAIT	Mucosal-associated invariant T
MDA-5	Melanoma differentiation associated 5
MHC	Major histocompatibility complex
MR1	MHC-related molecule 1
MMPs	Matrix metalloproteinases
MPO	Myeloperoxidase
MV	Measles virus
mTOR	Mammalian target of rapamycin
NCAM1	Neural cell adhesion molecule 1
NETs	Neutrophil extracellular traps
NF-κB	Nuclear factor kappa-light-chain-enhancer of activated B cells
PAMPs	Pathogen-associated molecular patterns
PD-L1	Programmed death-ligand 1
pDC	Plasmacytoid dendritic cells
PML	Promyelocytic leukaemia
pRB	Retinoblastoma protein
PRF1	Perforin 1
PVR	Poliovirus receptor
RIG-I	Retinoic acid inducible-gene I
SPP1	Secreted phosphoprotein 1 (also known as osteopontin)
STING	Stimulator of IFN genes
TCR	T cell receptor
T_CM_	Central memory T cell
T_EM_	Effector memory T cell
T_EMRA_	Terminally differentiated effector memory T cell
TGF-β	Transforming growth factor beta
Th1	T helper 1
TICAM1	TLR-receptor adaptor molecule 1
TIGIT	T cell immunoreceptor with Ig and ITIM (immunoreceptor tyrosine-based inhibitory motif) domains
TLRs	Toll-like receptors
TLSs	Tertiary lymphoid structures
T_MM_	Migratory memory T cell
TNF	tumour necrosis factor
T_RECIRC_	Recirculating T cell
Tregs	Regulatory T cells
T_RM_	Tissue-resident memory T cell
VIN	Vulvar intraepithelial neoplasia
VLP	Virus-like particle
